# Erythrocytic alpha-synuclein as potential biomarker for the differentiation between essential tremor and Parkinson’s disease

**DOI:** 10.3389/fneur.2023.1173074

**Published:** 2023-08-24

**Authors:** Zhenwei Yu, Genliang Liu, Yuanchu Zheng, Guoshi Huang, Tao Feng

**Affiliations:** ^1^Beijing Neurosurgical Institute, Capital Medical University, Beijing, China; ^2^Department of Neurology, Beijing Tiantan Hospital, Center for Movement Disorders, Capital Medical University, Beijing, China; ^3^China National Clinical Research Center for Neurological Diseases, Beijing, China

**Keywords:** essential tremor, Parkinson’s disease, alpha-synuclein, erythrocyte, biomarker

## Abstract

**Introduction:**

The differentiation between essential tremor (ET) and Parkinson’s disease (PD) can be difficult because of the symptom overlaps. Erythrocytes are the major source of peripheral α-synuclein (α-syn), which is the most studied pathological molecular of PD. We have reported that erythrocytic α-syn levels in PD patients are significantly increased compared to those in healthy controls (HCs). However, little is known about the levels of erythrocytic α-syn species in ET patients.

**Methods:**

This study includes 15 patients with ET, 64 patients with PD, and 49 age and sex matched HCs. A well-established electrochemiluminescence assay was used to measure the erythrocytic total and aggregated α-syn levels. The receiver operating characteristic (ROC) curve analysis was applied to evaluate the diagnostic values of erythrocytic α-syn for ET diagnosis and differentiation. The correlations of erythrocytic α-syn levels with disease durations were tested using Spearman’s Rank Correlation analysis.

**Results:**

We found that both erythrocytic total and aggregated α-syn concentrations are significantly increased in PD and ET patients compared to those in HCs. Erythrocytic total α-syn levels are significantly higher in ET patients than those in PD group. Furthermore, the ratios of erythrocytic aggregated to total α-syn levels in ET patients are significantly decreased than those in PD and HC subjects. We also found a significant association of erythrocytic aggregated α-syn levels with the disease duration of ET patients.

**Conclusion:**

Our findings suggest new insight into the changes of erythrocytic total and aggregated α-syn levels as potential biomarkers for ET patients.

## Introduction

Parkinson’s disease (PD) and essential tremor (ET) are the most prevalent movement disorders in the elderly (1). Misdiagnoses of tremulous Parkinson’s disease (PD) and essential tremor (ET) are very prevalent, in part because tremulous PD might appear as monosymptomatic tremor at the early stage and advanced ET may proceed into the resting condition (2–4). Actually, rest tremor, a key feature of PD, is also present in 18.8% of ET patients (2).

According to earlier research, nearly half of the ET cases were misdiagnosed with PD or other types of tremors (5). There is a critical need for efficient diagnostic biomarkers for ET.

The most studied pathological biomarkers for PD are alpha-synuclein (α-syn) and its aggregated variants (6). However, the role α-syn plays in the pathogenesis of ET is unclear. Esther et al. demonstrated a decrease in plasma α-syn levels in PD and ET compared to HCs, which was independently verified by another group (7). Additionally, Lewy bodies, the pathological hallmark of PD which contains α-syn, have been discovered in certain postmortem examinations of ET (8). However, the risk of ET is not correlated with variants in the SNCA locus according to a meta-analysis including 661 ET subjects and 1,316 controls (9). Recently, we have demonstrated that monomeric and aggregated α-syn levels in erythrocytes are significantly increased in PD patients compared to those in healthy controls (HCs) (10). However, the performance of these biomarkers for ET diagnosis and differentiation with PD has not been reported before.

In the present study, we aim to study the diagnostic and differentiative value of erythrocytic α-syn concentrations for ET and the correlations with clinical characteristics.

## Materials and methods

### Demographic characteristics of participants

63 idiopathic PD patients, 15 ET patients, and 49 age and sex matched HCs were recruited from Beijing Tiantan Hospital in the current study. All PD and ET patients were diagnosed according to the Movement Disorder Society Clinical Diagnostic Criteria (11, 12), with the exclusion criteria including atypical or secondary PD syndrome, severe head injury, stroke history, severe psychiatric disorders, and severe systemic disorders. The exclusion criteria for healthy controls includes a diagnosis of any movement disorders or other neurological diseases. The exclusion criteria for ET patients includes other neurologic signs, such as dystonia, ataxia, or parkinsonism.

The clinical and demographic data including age, sex, and disease duration were gathered and presented in Table 1 for all participants, with informed consent obtained. This study was reviewed and approved by the Ethics Committee of Beijing Tiantan Hospital, Capital Medical University.

**Table 1:** Demographic information.

Group	Healthy controls	Parkinson’s disease	Essential tremor	*p*-value	*p*-value	*p*-value
				HC vs. PD	HC vs. ET	PD vs. ET
Number	49	64	15	NA	NA	NA
Gender (male: female)	26: 23	34:30	8: 7	*p* = 0.995	*p* = 0.985	*p* = 0.988
Age (median, range)	61 (48–79)	60 (29–82)	64 (38–77)	*p* > 0.999	*p* > 0.999	*p* > 0.999
Disease duration (median, range)	NA	7 (0.5–16)	8 (1–20)	NA	NA	*p* = 0.900

### Sample preparations and measurements

Polypropylene collection and storage tubes containing EDTA (BD Biosciences, CA, United States) were used to collect the venous blood of all participants in the morning after 12 h starvation. The erythrocytes were in the pellet after centrifuging at 4°C and 2,000 g for 15 min. Then the erythrocytes were transferred to a new tube and mixed with pre-chilled STET buffer (pH 8.0; Leagene, Beijing, China) at a 1:100 ratio. The mixtures were vortexed for 15 s, rotated at 4°C for 30 min, and then centrifuged at 4°C 12,000 g for 10 min to remove the cell debris. The supernatant was preserved for aggregated α-syn measurement, and the samples were further diluted in STET buffer at a 1:100 ratio for monomeric α-syn measurement.

The quantification of erythrocyte aggregated and monomeric α-syn was characterized using a home-brewed 96-well Meso Scale Discovery (MSD, Rockville, MD, United States) U-Plex assay, which has been described previously (10). Briefly, biotinylated capture antibodies (ab138501, ab209538, Abcam, Cambridge, MA, United States) were coated to the MSD U-Plex plate, and the excess antibodies were washed off by three times wash using 150 μL 1 × wash buffer (MSD, Rockville, MD, United States). Protein standards were purchased from Proteos (Alpha-synuclein Protein-monomer; Cat# PR-001, Alpha-synuclein Protein-filament; Cat# PR-002, Proteos, Inc.) and loaded together with samples to the capture antibody-coated U-Plex plate for 1 h with continuous 600 rpm shaking. Then the plate was washed 3 times with 150 μL 1 × wash buffer (MSD, Rockville, MD, United States), and coated with 50 μL Sulfo-tagged detection antibody (610,786, BD Bioscience, CA, United States) for 1 h with 600 rpm shaking. After 3 times washing again, 150 μL 2 × Read Buffer T (MSD, Rockville, MD, United States) was applied to the wells before plate reading on MSD QuickPlex SQ120 for protein quantification.

### Statistical analysis

GraphPad Prism 9 (GraphPad Software, La Jolla, CA, United States) and IBM SPSS 25 (IBM, Chicago, IL, United States) were used for the statistical analysis. The concentrations of erythrocytic monomeric and aggregated α-syn were normalized to the volume of the erythrocyte. The normality was analyzed using the Kolmogorov–Smirnov test, and the Kruskal-Wallis test with Dunn’s multiple tests was applied for three groups comparison. The correlation between biomarkers and disease duration was analyzed using Spearman’s rank test. The area under curve (AUC) was determined using the receiver operating characteristic (ROC) curve, with the best sensitivity and specificity determined using the Youden index. *p* < 0.05 was considered significant.

## Results

In the current study, we included a total of 127 samples and clinical characteristics of subjects. In brief, 63 patients with PD with a disease duration ranging from 0.5 to 16 years, and 15 ET patients with disease duration from 1 to 20 years together with 49 age and sex matched healthy controls were recruited. The demographic information and clinical characteristics were listed in Table 1.

**Figure 1:**
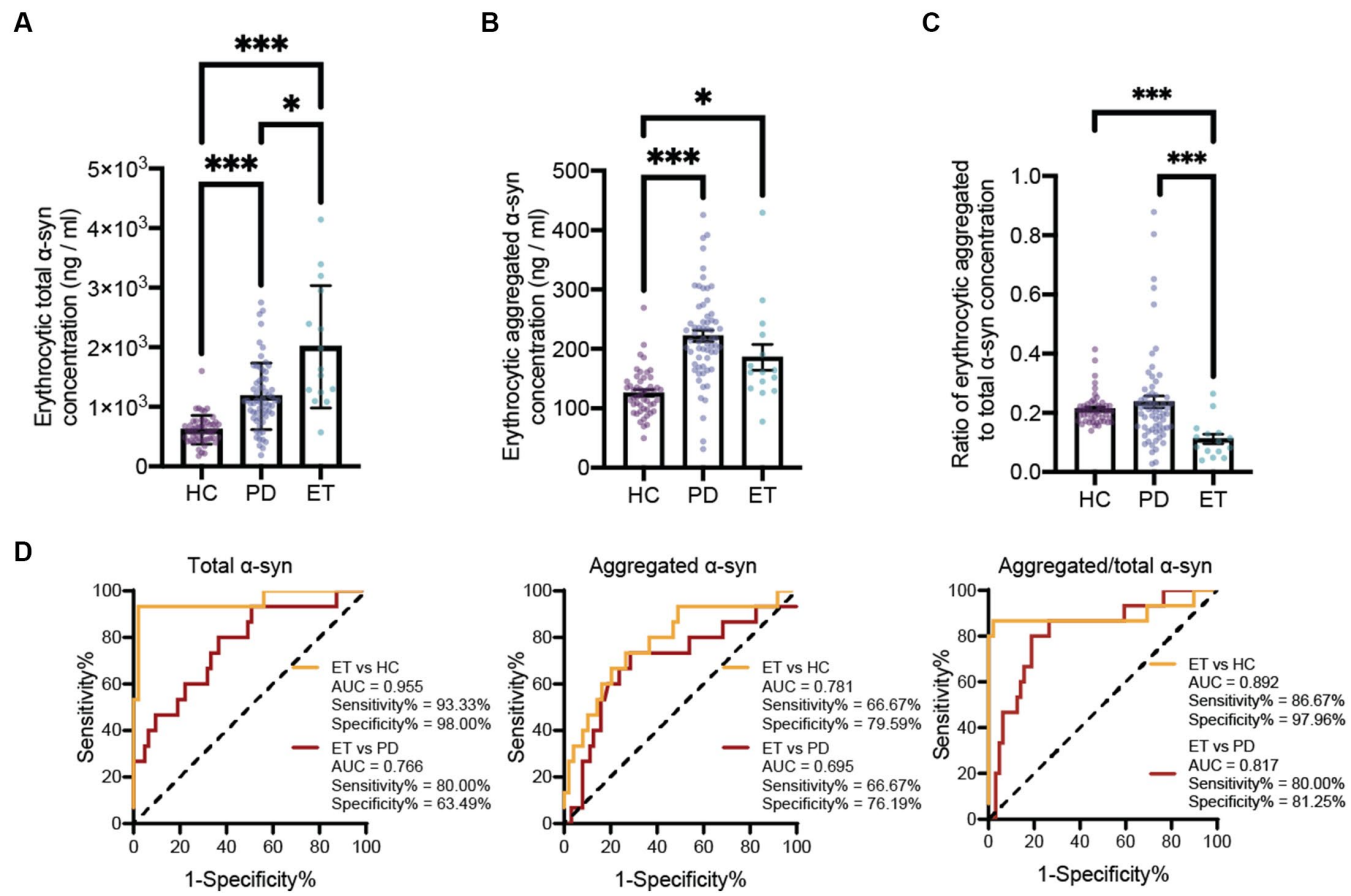
Evaluation of erythrocytic α-syn levels and the ROC curve analysis for the ratio of aggregated to total α-syn to differentiate ET patients from PD patients and HCs. Assessment of erythrocytic total **(A)** and aggregated **(B)** α-syn levels. ^*^p < 0.05, ^***^p < 0.001. **(C)** Assessment of the ratio of erythrocytic aggregated to total α-syn levels. ^*^p < 0.05, ^***^p < 0.001. **(D)** Receiver operating characteristic curve for the erythrocytic total α-syn, erythrocytic aggregated α-syn and the ratio of erythrocytic aggregated to total α-syn levels in differentiate ET patients from PD patients and HCs. ET, essential tremor; HC, healthy control; PD, Parkinson’s disease; AUC, area under curve; α-syn, α-synuclein.

The results demonstrated statistically significant increases in monomeric α-syn concentrations in erythrocytes of PD and ET patients compared to those in HCs. Besides, the erythrocytic monomeric α-syn concentrations in the ET group are also significantly higher than those in PD patients (Figure 1A). The aggregated form of α-syn concentrations in erythrocytes of PD and ET patients are also significantly increased than those in HCs. Although no statistically significant difference was found between the erythrocytic aggregated α-syn concentrations of PD and ET patients, there is a decreasing trend in ET subjects (Figure 1B). Interestingly, there is no statistical difference between the ratios of aggregated α-syn to monomeric α-syn concentrations between PD and HC subjects. However, the ratios of aggregated α-syn to monomeric α-syn concentrations are significantly lower in ET subjects than those in both PD and HC groups (Figure 1C). Receiver Operation Characteristic (ROC) curves analysis demonstrated high accuracy for the ratios of aggregated α-syn to monomeric α-syn concentrations in discriminating ET from PD and HC subjects, with the AUC 0.892, sensitivity% 86.67, specificity% 97.96 for ET vs. HC, and AUC 0.817, sensitivity% 80.00, specificity% 81.25 for ET vs. PD (Figure 1D). Notably, the ratios of aggregated α-syn to monomeric α-syn concentrations do not discriminate PD from HC significantly, but both erythrocytic aggregated and monomeric α-syn concentrations are good diagnostic biomarkers for PD (aggregated α-syn concentration: AUC = 0.893, sensitivity% = 82.54%, specificity% = 89.80%; monomeric α-syn concentration: AUC = 0.841, sensitivity% = 79.37%, specificity% = 86.00%, Supplementary Figure S1), which is consistence with previous reports (10).

**Figure 2:**
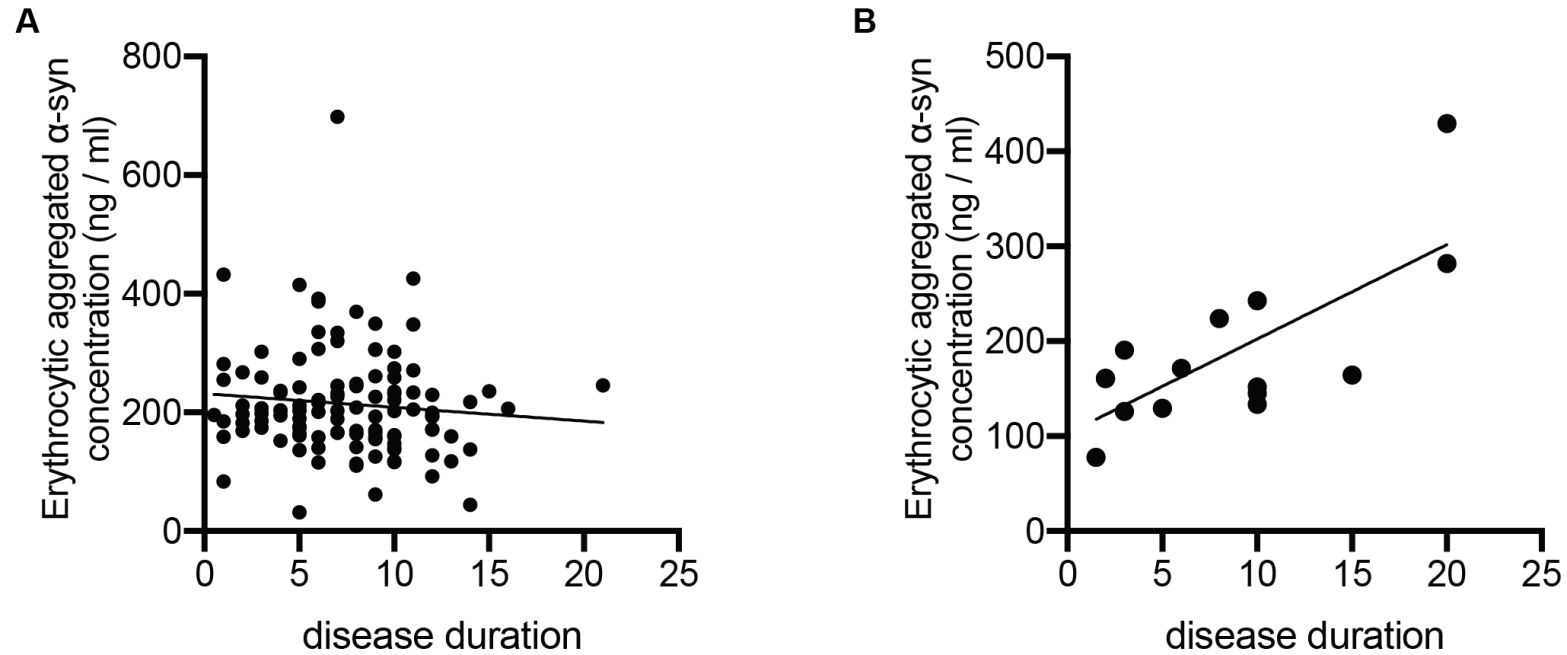
Correlation analysis of erythrocytic aggregated α-syn levels with disease durations of PD and ET patients. **(A)** No significant correlations between erythrocytic aggregated α-syn concentrations and disease durations (*p* = 0.998, r < 0.001) were observed in PD patients. **(B)** Erythrocytic aggregated α-syn concentrations were significantly correlated with disease durations (*p* = 0.036, r = 0.551) in ET patients. ET, essential tremor; PD, Parkinson’s disease; α-syn, α-synuclein.

To further study the potential change of erythrocytic α-syn concentrations in accordance with the disease course of ET and PD, we calculated the correlations of both aggregated and monomeric α-syn concentrations with disease durations of ET and PD patients. Notably, aggregated α-syn values are significantly and positively related to disease duration of ET (*p* < 0.01; Figure 2B), but not PD patients (*p* = 0.99; Figure 2A). However, no significant correlations of monomeric α-syn values and disease durations of ET (*p* = 0.12) or PD (*p* = 0.72) patients were found (data not presented).

## Discussion

In the present study, we demonstrate that the monomeric and aggregated α-syn levels in erythrocytes of ET patients are significantly increased than HCs, and the ratio of aggregated to monomeric α-syn concentrations are significantly decreased in ET subjects compared to those in PD and HC subjects. The erythrocytic α-syn species concentrations are potential diagnostic and differential biomarkers for ET patients with high accuracy. Furthermore, the erythrocytic aggregated α-syn concentrations are positively correlated with the disease duration of ET but not PD patients.

The detection of pathological proteins such as α-syn and its variants in bio-specimen constitutes a valuable method for identifying and differentiating α-synucleinopathies including PD (13). Although the pathological α-syn aggregations mainly localize in the central nervous system, peripheral α-syn concentrations, especially those in erythrocytes are significantly higher than those in cerebrospinal fluid (CSF) (14, 15). Recently, we have reported that erythrocytic α-syn species are significantly increased in PD and multiple system atrophy (MSA) subjects compared to HCs. Junichi et al. reported that α-syn-containing erythrocyte-derived EVs can pass through the blood–brain barrier from peripheral blood to the central nervous system (16). Increasing evidence supports the theory that erythrocytes change is critical in the pathogenesis of α-syn related neurodegenerative diseases. However, there are few studies demonstrating the role of α-syn implicated in ET, a common movement disorder that is frequently misdiagnosed as PD. It has been widely reported that a proportion of ET patients (20%) will finally develop PD, which is characterized as ET-PD (17). There are also ET-plus patients associated with parkinsonism, which is controversial and makes it difficult to distinguish those patients with PD according to clinical features (18, 19). So, in the current study, we only recruited ET patients without any signs of neurologic symptoms such as dystonia, ataxia, or parkinsonism to eliminate potential distractions. However, a DaT PET scan could significantly benefit the participant selection for ET patients to eliminate the potential ET-PD patients that have not exhibits parkinsonian symptoms. Although about 20% of ET patients exhibit Lewy bodies in the brain (20), ET is not generally considered as a synucleinopathy disease. However, our data suggest an increase of both aggregated and monomeric α-syn concentrations in erythrocytes of ET patients than HCs, supporting the potential implication of α-syn in ET pathology. As far as we know, this is the first research to demonstrate levels of erythrocytic α-syn in ET patients. Interestingly, although there are no significant differences between both the erythrocytic aggregated and monomeric α-syn concentrations between PD and ET patients, the ratios of aggregated α-syn to monomeric α-syn concentrations can significantly differentiate ET patients from PD and HCs. Silvia et al. recently reported a decrease in plasma α-syn levels in PD and ET patients compared to HCs, but there are no significant differences between ET and PD (OFF, ON, *de novo*-PD) patients (7). One possible explanation is that pathological α-syn accumulates in erythrocytes, which reduces the free α-syn released to plasma. There is continuous debate about the relationship between ET and PD due to the overlapping motor features and genetic mutations, but it is generally accepted that these are two distinct clinical entities. Our data support this statement since the ratio of aggregated α-syn to monomeric α-syn concentrations in ET are significantly lower than in PD patients. However, uncertainty surrounds the pathogenic significance of this conversion of erythrocytic aggregated α-syn to monomers in ET, which deserves further study.

We also observed a significant positive correlation of erythrocytic aggregated α-syn concentrations with disease duration of ET but not PD patients. Although some studies demonstrated the α-syn pathology associated with ET, the mechanism of action is largely unknown. The current results support the hypothesis that ET and PD have distinct pathological mechanisms. Additionally, the levels of aggregated α-syn in erythrocytes may serve as a good biomarker for the indication of ET disease course. However, the sample size is small, especially for ET subjects, which is a significant limitation of this study. The results should be verified in a large cohort in the future. Furthermore, whether these ET-related biomarkers can predict the disease prognostics is still unknown, which calls for more research and patient enrollment with follow-up visits.

In conclusion, erythrocytic aggregated and monomeric α-syn levels and their ratios are valuable biomarkers that differentiate ET patients from PD and HCs. The higher erythrocytic aggregated α-syn concentrations are in accordance with the disease duration of ET but not PD patients.

## Data availability statement

The raw data supporting the conclusions of this article will be made available by the authors, without undue reservation.

## Ethics statement

The studies involving humans were approved by the Ethics Committee of Beijing Tiantan Hospital. The studies were conducted in accordance with the local legislation and institutional requirements. The participants provided their written informed consent to participate in this study.

## Author contributions

TF and YZ designed the study. ZY and YZ performed the measurements, data analysis, and wrote the manuscript. GH contributed to the data analysis. GL contributed to sample collection and preparation. All authors contributed to the article and approved the submitted version.

## Funding

This work was funded by the National Nature Science Foundation of China (nos. 81771367, 82071422, and 82020108012) and Beijing Municipal Natural Science Foundation (nos. 7212031 and 7232013).

## Conflict of interest

The authors declare that the research was conducted in the absence of any commercial or financial relationships that could be construed as a potential conflict of interest.

## Publisher’s note

All claims expressed in this article are solely those of the authors and do not necessarily represent those of their affiliated organizations, or those of the publisher, the editors and the reviewers. Any product that may be evaluated in this article, or claim that may be made by its manufacturer, is not guaranteed or endorsed by the publisher.

## Supplementary material

SUPPLEMENTARY FIGURE S1 Receiver operating characteristic curve for the ratio of erythrocytic aggregated and total α-syn levels to differentiate PD patients from HCs. HC, healthy control; PD, Parkinson’s disease; AUC, area under curve; α-syn, α-synuclein.
