# Supplementary figures and images for: Erythrocytic alpha-synuclein as potential biomarker for the differentiation between essential tremor and Parkinson’s disease
Source: Front Neurol. 2023 Aug 24;14:1173074. doi: 10.3389/fneur.2023.1173074 (PMC10483808; doi:10.3389/fneur.2023.1173074)

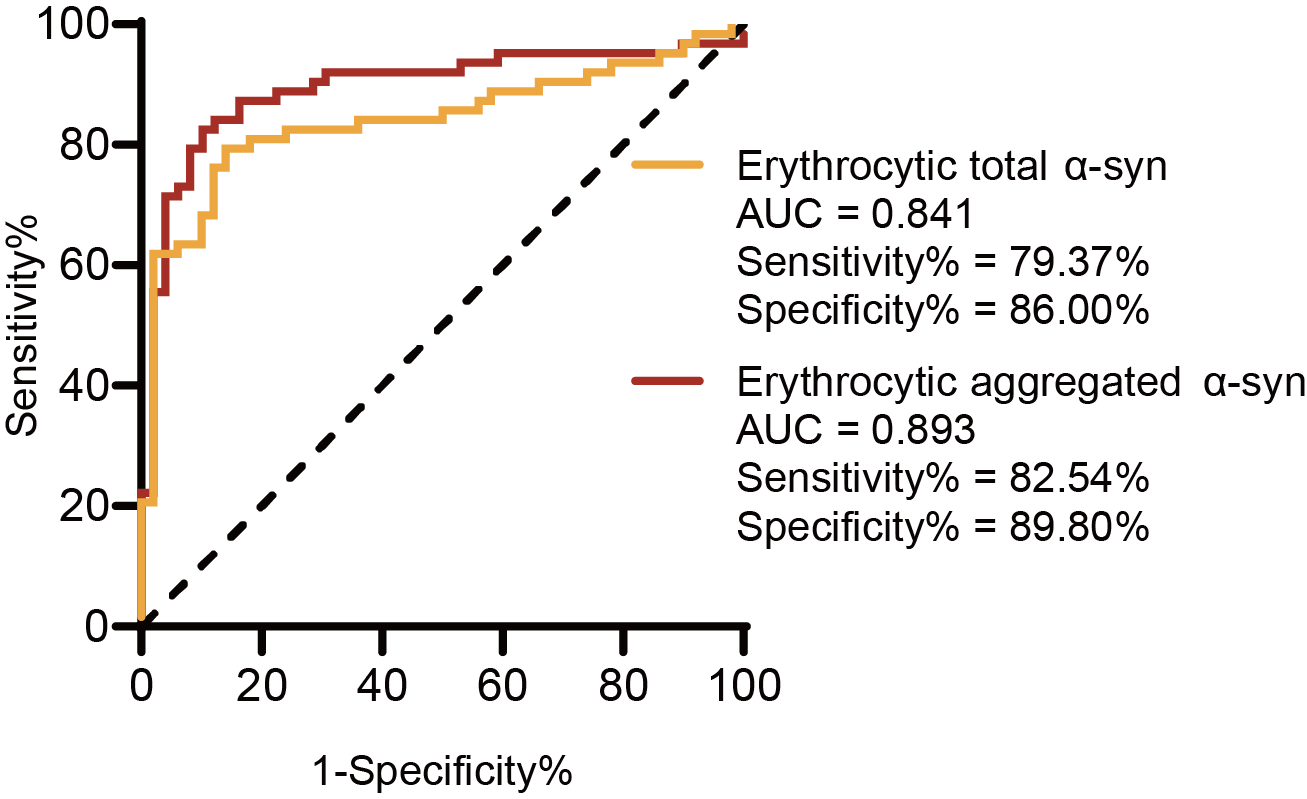

Supplement: SUPPLEMENTARY FIGURE S1 — Receiver operating characteristic curve for the ratio of erythrocytic aggregated and total α-syn levels to differentiate PD patients from HCs. HC, healthy control; PD, Parkinson’s disease; AUC, area under curve; α-syn, α-synuclein. [file Image_1.TIF]
